# Inter-Individual Differences in Food Addiction and Other Forms of Addictive-Like Eating Behavior

**DOI:** 10.3390/nu13020325

**Published:** 2021-01-23

**Authors:** Paul Brunault, Nicolas Ballon

**Affiliations:** 1 UMR 1253, iBrain, Université de Tours, Inserm, 37000 Tours, France; nicolas.ballon@univ-tours.fr; 2 CHRU de Tours, Service d’Addictologie Universitaire, Équipe de Liaison et de Soins en Addictologie, 37000 Tours, France; 3 Qualipsy EE 1901, Université de Tours, 37000 Tours, France

The “addictive-like eating behavior” phenotype encompasses different terms or concepts, including “food addiction” (FA), “eating addiction” or “compulsive eating behavior” [1,2,3]. Although these terms may theoretically refer to different conceptualizations of addictive-like eating, all agree on the similarities this phenotype may share with other addictive disorders in terms of diagnostic criteria (with some core symptoms being food craving, loss of control over eating, and maintenance of the behavior despite negative consequences), epidemiology, risk factors, and treatment [1,2,3]. The main hypothesis underlying the “addictive-like eating behavior” phenotype is that it may help identify, among persons with obesity, eating disorders or persons with other eating symptoms, a specific and distinct subpopulation of vulnerable individuals for whom specific therapeutic management strategies may be proposed [4]. Although the FA model has the potential to open new avenues of conceptualization and management in obesity and eating disorders by providing new options to the existing treatments [3,5], some authors questions the validity and the specificity of the FA/addictive-like eating behavior phenotype [6,7]. To explain the possible inconsistencies in this evidence, and to gain insight into the possible validity and clinical utility of the addictive-like eating behavior phenotype, we argue here that we have to take into account the heterogeneous nature of FA. Beyond the identification of this phenotype, we hypothesize here that one key issue may be, as already demonstrated for other addictive disorders, that different psychobiological factors or different pathways may account for this increased vulnerability, with the identification of different clusters of vulnerable patients [8,9,10,11]. By examining the inter-individual differences that could account for this phenotype, and by disentangling the contribution of these different factors for a given individual, we may then propose tailor-based interventions based on the specific psychobiological factors involved. Before directly testing this hypothesis in interventional studies, one preliminary step is to identify what are the psychobiological factors associated with this “addictive-like eating behavior” phenotype in different clinical and non-clinical populations, and to determine how these factors may cluster together to help identify these different clusters of patients.

The aim of the special issue is to provide a modest—but hopefully constructive—contribution to this research area, by presenting research studies and literature reviews that will improve our understanding of the “addictive-like eating behavior” phenotype and of its associated factors, thus paving the way for the identification of these clusters of patients. This special issue includes eleven studies (four reviews and seven original studies). These seven original studies investigate the factors associated with this phenotype in different contexts: patients seeking treatment for an eating disorder, including anorexia nervosa, bulimia nervosa or binge eating disorder [12,13], patients seeking treatment for obesity (i.e., bariatric surgery candidates) [14], and non-clinical populations (i.e., persons not directly seeking treatment for these conditions) such as adolescents [15], female restrained eaters [16], and persons with weight-related disorders recruited in the general population [17]. This special issue also present four literature reviews that focus on: neuroimaging of sex/gender differences in obesity, in which FA is prevalent [18], involvement of the melanocortin system in binge eating, food reward and motivation [19], association between addictive-like eating behavior and attention-deficit/hyperactivity disorder (ADHD) [20], and a discussion about the FA concept and its practical implications through four complementary disciplines: addiction medicine, nutrition, health psychology, and behavioral neuroscience [21].

Obesity is the most studied population in the FA research field because of their high co-occurrence [22]. Benzerouk et al. focused on the association between binge eating, emotion dysregulation, impulsivity, depression, and anxiety in bariatric surgery candidates [14]. They confirmed that persons at risk for binge eating disorder were more prone to report limited access to emotion regulation strategies as well as higher impulsivity. More importantly, they demonstrated in multivariable models that emotional eating, external eating, and binge eating were independently associated with specific dimensions of emotion regulation and impulsivity. Although treatment-seeking persons with obesity are more frequently women, obesity is also prevalent in men. When studying the inter-individual differences, gender/sex may be an important factor to consider. In an elegant literature review, Kroll et al. shed light on the neuroimaging of sex/gender differences in persons with obesity, with a focus on structure, function, and neurotransmission [18]. They highlighted inter-individual differences based on gender: changes in somatosensory regions appeared to be associated with obesity in men, while changes in reward regions were more strongly associated with obesity in women than in men. They also found sex/gender differences in the neural response to taste among persons with obesity. These data are consistent with the idea that different neural mechanisms may be observed in obesity, and that the consideration of gender/sex may help in designing more tailored interventions based on the specific mechanisms involved. Future studies could investigate these interesting inter-individual differences in persons with obesity and binge eating disorder/FA vs. persons with obesity but without disordered eating.

To demonstrate the reliability and the relevance of the FA phenotype, one complementary area for research focusses on the biological correlates of FA in persons with obesity or overweight. In the FA field, fewer studies have been conducted using biobehavioral or genetic measures than subjective measures. To advance knowledge in this field, Aviram-Friedeman compared the brain asymmetry at rest and cue-reactivity to images of rewarding food in a Stroop Task between persons with weight-related problems and FA (group 1), persons with weight-related problems but no FA (group 2), and persons without weight-related problems and no FA (control group). The group with overweight/obesity and FA displayed a specific profile that was not observed in the two other groups: a lower resting left alpha brain asymmetry, an attenuated Stroop bias following exposure to high-calorie food relative to nonfood image, and a lower late positive potential component in frontal and occipital regions. Although association does not mean causation, these results point out the possibility of neural correlates specific to FA, as well as the need to consider how environmental stimuli may trigger or exacerbate FA. As demonstrated for addictive disorders, addictive-like eating may result from a gene-environment interaction, and Micioni Di Bonaventura et al. provide here a focused review of how alterations in the melanocortin system may be involved in obesity, binge eating behavior, and food reward/motivation [19]. In this literature review based on preclinical and clinical studies, these authors underline the pivotal role of the melanocortin system in controlling feeding behavior, appetite, energy balance, motivation for highly palatable food, and stress regulation. They discuss here how the loss of function of the melanocortin receptors (especially through the genetic variations of the MC3R and the MC4R, that are mainly located in the mesolimbic dopamine system) may lead to a breakdown of normal regulatory processes, thus increasing the risk of overeating and binge eating.

In addition to patients with obesity, patients with eating disorders are also persons for whom the FA phenotype may be useful. Fewer studies have been conducted in clinical persons seeking treatment for an eating disorder than in persons with obesity, especially in samples of persons with the full spectrum of eating disorders [22]. In a large sample of 195 adult women referred to an eating disorder treatment center for anorexia nervosa, bulimia nervosa, or binge eating disorder, Fauconnier et al. assessed the prevalence of FA and its associated factors [12]. In line with Granero [23], they confirmed the high prevalence of the FA phenotype in all types of eating disorders, with prevalence rates being respectively 61.5% for anorexia nervosa restrictive subtype, 87.9% for anorexia nervosa binge-eating/purging subtype, 93.3% for binge eating disorder, and 97.6% for bulimia nervosa. The main study result was the demonstration that the FA phenotype was independently associated with three variables: the presence of recurrent episodes of binge eating, eating disorder severity, and lower interoceptive awareness (this latter being compatible with one aspect of the three-systems neural model of addiction proposed by Noël et al., with the involvement of the insula in interoceptive awareness [10]). In another study presented here, Bou Khalil et al. tested among persons seeking treatment for an eating disorder whether FA was associated with a more severe eating disorder symptom severity and with more frequent childhood maltreatment, with the hypothesis that FA may mediate the relationship between childhood trauma and eating disorder severity [13]. They found that existence of FA was associated with a more severe eating disorder and with all types of traumas. Their data were compatible with a mediational role of FA in the relationship between childhood trauma and eating disorder severity, with largest effects emerging for physical neglect and emotional abuse. These data strengthen the hypothesis that the identification of a FA phenotype among eating disorder patients may help in identifying a distinct subpopulation of vulnerable individuals for whom specific therapeutic management strategies could be proposed, with low interoceptive awareness and childhood trauma being two potential targets. More studies in this specific population will be helpful to test (and eventually refine) this hypothesis, especially using interventional studies. 

One potential target for the treatment of persons with FA is emotional eating and emotion dysregulation. Emotion dysregulation is indeed strongly associated with addictive disorders, and it is now integrated as a core component of addictive disorders treatment. In a large sample of 1142 persons recruited in the general population, Bourdier et al. used correlation and mediation analyses to compare the association between anxiety, depression, FA, emotional eating and the difficulty to rely on hunger and satiety cues between persons with versus without obesity [17]. First, they found associations between depression, anxiety, FA symptoms and the difficulty in relying on hunger and satiety cues across all weight classes. They also examined the association between emotional eating and these factors in each weight class, and they further demonstrated that emotional eating was associated with these factors but only in persons with obesity. Emotional eating may be an important factor to consider in persons with obesity: data were compatible with a meditational role of negative emotional eating in the relationship between anxiety symptoms and the difficulty in relying on internal cues to regulate food intake, as well as between depression symptoms and the difficulty in relying on these internal cues. Emotion dysregulation is also a core component of some psychiatric disorders, especially ADHD. ADHD symptoms and diagnosis are associated with addictive disorders and eating disorders, but the exact mechanisms underlying this association are currently unclear. In a literature review that included 41 papers, El Archi et al. report that ADHD and disordered eating are significantly associated, especially for binge eating and addictive-like eating behavior/FA. This review supports the idea that negative affectivity and emotion dysregulation may be mediators in the relationship between ADHD and disordered eating, providing another potential treatment option for persons with addictive-like eating behavior. As emotion dysregulation is prevalent in persons with psychiatric disorders, targeting emotion dysregulation may be especially relevant in these persons with addictive-like eating behavior and a co-occurring psychiatric disorder. 

The addictive like-eating behavior phenotype may also be useful outside the obesity and eating disorders fields. Adolescence is an at-risk population for addictive disorders, given the higher impulsivity and less efficient emotional regulation processes compared to adults. In a controlled study, Rodrigue et al. examine the cognitive factors associated with FA symptoms in adolescence [15]. They compare adolescents with versus without FA symptoms in terms of “objective” sustained attention and executive functions (CANTAB neuropsychological battery) and in terms of “subjective” assessment (self-reported questionnaire assessing executive functions). Interestingly, adolescents with FA symptoms had higher subjective executive difficulties (as assessed by the self-reported questionnaires) than adolescents without FA, but there was no difference in terms of “objective” executive functions (as assessed by the neuropsychological tasks). When studying potential risk factors for FA in adolescents, the authors suggest the assessment of subjective executive difficulties (rather than only the objective ones) in addition to impulsivity, depression and anxiety. In addition to adolescents, persons with restrained eating may also be at risk for FA. As hypothesized by Herman & Polivy’s theory and Fairburn et al.’s cognitive-behavioral model of bulimia nervosa, loss of control over food intake may arise from dietary/cognitive restraint (i.e., an intentional effort to achieve weight loss through reduced caloric intake) [24]. In a two-fold randomized-controlled study, Weinbach et al. assessed among female restrained eaters the impact of two different response inhibition trainings on food consumption, food related anxiety, and implicit attitudes toward food [16]. They compared two types of responses: a food-response training, in which stop cues were always associated with non-food images, and a balanced food-response/inhibition training, in which participants inhibited motor actions to food and non-food stimuli equally. Contrary to the food-response group, participants in the food-response/inhibition training group reduced their snack consumption and experienced an increase in positive attitudes toward palatable foods. Cognitive training may be helpful in restrained eaters, and future studies could investigate its effects on eating behavior and weight in clinical population with FA and restrained eating. 

A comprehensive understanding of the factors associated with the addictive-like eating behavior phenotype is a preliminary step towards tailor-based treatments. In a review published in this special issue, Constant et al. argues in favor of a multidisciplinary dialogue between specialists in addiction medicine, clinical nutrition, health psychology, and behavioral neuroscience to account for and manage the complexity of FA [21]. After the presentation of each specialist’s point of view as well as a literature review in each field, they proposed a multidisciplinary framework and practical implications derived from this framework in order to improve FA prevention, diagnosis, and treatment in at-risk populations. They proposed that only a multidisciplinary perspective can render the complexity of FA and how it relates to environmental, social, and individual factors, with FA being considered in a continuum ranging from normal to disordered eating. 

Altogether, the studies gathered in this special issue support the idea that the “addictive-like eating behavior” phenotype, especially FA, may help in identifying a specific subpopulation of vulnerable individuals (i.e., persons with this phenotype differ from those without in different psychobiological factors). Addictive-like eating behavior was indeed associated across different populations with different psychobiological factors, including emotion dysregulation, higher prevalence for childhood traumas, lower emotional awareness, anxiety and depressive symptoms, difficulties to rely on internal cues, but also EEG abnormalities or alterations in the melanocortin system. Future challenges will be to address the questions of the validity and of the clinical utility of addictive-like eating behavior (i.e., how these different and specific psychobiological factors may cluster together to identify different subsets of vulnerable patients; how results from this field of research should be integrated into the existing models of obesity and/or eating disorders to provide treatment advances). To achieve this aim, we argue here that we need to take into account the inter-individual differences in addictive-like eating behavior. Studies conducted outside the FA field have already provided valuable insights into the inter-individual differences in addictive disorders [8,9,10,11], and these theoretical models may be useful to address the complexity of the FA puzzle and help in designing better tailor-based treatment for these persons. 
